# Beyond admission scores: evaluating GAT, SAAT, and English proficiency as predictors of physics performance in health science education

**DOI:** 10.3389/fmed.2025.1595079

**Published:** 2025-07-08

**Authors:** Abdulaziz Althewini

**Affiliations:** King Saud bin Abdulaziz University for Health Sciences, King Abdullah International Medical Research Center and Ministry of National Guard - Health Affairs, Riyadh, Saudi Arabia

**Keywords:** college admissions, standardized tests, physics performance, English proficiency, predictive validity

## Abstract

**Background:**

Accurate prediction of academic performance is essential for selecting students into competitive medical and health science programs. In Saudi Arabia, standardized cognitive assessments—such as the General Aptitude Test (GAT) and Scholastic Achievement Admission Test (SAAT)—are widely used for admissions. This study evaluates the predictive validity of these tests, alongside English proficiency measures, in forecasting student performance in an introductory physics course—a foundational subject in science-based programs.

**Methods:**

This retrospective, quantitative study analyzed data from 250 Saudi college students enrolled in a required introductory physics course. Predictor variables included GAT scores (critical thinking and reasoning), SAAT scores (content knowledge in science and math), and English proficiency, assessed via three metrics: preparatory-year English course average, reading test scores, and communication skills test scores. Both simple linear regression and multiple regression analyses were conducted to evaluate the individual and combined predictive contributions of these variables to final physics course grades.

**Results:**

All predictors were statistically significant. Among them, reading proficiency was the strongest individual predictor, accounting for 19.6% of the variance in physics grades, followed by GAT (9.4%) and SAAT (7.9%). Communication test scores explained a smaller portion (7.2%). The combined model explained 29.3% of the total variance in physics performance, leaving approximately 70% of the variance unexplained by the selected cognitive measures.

**Conclusion:**

Although GAT, SAAT, and English reading proficiency contribute modestly to predicting physics course performance, their limited combined predictive power points to the need for more comprehensive admissions criteria. Non-cognitive factors—such as motivation, study habits, or self-efficacy—may significantly influence academic outcomes but remain unmeasured in current systems. These findings support calls for reforming admissions practices in Saudi health science education to adopt a more holistic and evidence-informed approach.

## 1 Introduction

The selection of students for medical and health science programs is a critical process that shapes the future of healthcare professionals. Pre-admission criteria aim to identify candidates most likely to succeed academically and professionally in these demanding fields (1–3). These typically include high school grades and standardized tests that assess analytical, deductive, and problem-solving abilities. The relative weight assigned to each criterion varies across institutions, yet with the increasing number of applicants, ensuring the robustness and relevance of these criteria is more pressing than ever (3–7). However, few studies have isolated performance in specific foundational science courses—such as physics—as an early indicator of academic readiness in medical and health science education.

In Saudi Arabia, admission to medical and health science colleges typically relies on a combination of high school grades, the Scholastic Achievement Admission Test (SAAT), the General Aptitude Test (GAT), and English proficiency attained during the preparatory year. Although these criteria are designed to identify students with strong academic potential, their ability to accurately predict success remains debated. Some studies have reported meaningful correlations between these measures and early academic performance, particularly during the pre-clinical stages of medical education (8–11). However, other research has found these associations to be weak or inconsistent, especially as students advance into clinical phases where non-cognitive skills—such as clinical reasoning and communication—become more critical (12, 13).

Although standardized admission tools such as the GAT and SAAT remain central to student selection processes in Saudi Arabia, their predictive value remains inconclusive. A growing body of global and Saudi research has examined the effectiveness of these assessments, but the findings are often inconsistent and lack contextual specificity (14, 15, 31). A key limitation is the frequent reliance on broad academic outcomes as the primary measure of success, which may mask variations in performance across different subject areas. Relatively few studies have investigated how these admission criteria relate to success in specific science courses like physics, which require both cognitive rigor and strong language skills. In particular, English language proficiency—especially reading comprehension—is often underemphasized, despite its critical role in navigating complex scientific content in English-medium programs (16, 17). These gaps underscore the need for more focused research that integrates both cognitive and language-related predictors to better assess academic readiness in health science education.

Moreover, many local studies have evaluated academic performance primarily through standardized test outcomes such as GAT and SAAT scores (9, 18–20). While these tests offer useful indicators of academic aptitude, they may fail to capture the full range of competencies required for success in modern medical education. Key skills such as academic language proficiency, clinical reasoning, teamwork, and professional communication—now central to competency-based medical education (CBME) models—are often overlooked in traditional assessments (21, 22). Furthermore, with increasing global attention on holistic admissions, there is growing recognition that predictive models should account for both cognitive and non-cognitive dimensions of student potential (23, 24). This perspective calls for broader, evidence-based frameworks that reflect the complexity of academic and professional success in health sciences.

In framing this investigation, two theoretical perspectives are particularly relevant. First, Messick’s (32) unified theory of validity provides a foundation for evaluating the predictive function of admission tests, emphasizing that validity must reflect the extent to which assessment scores support meaningful inferences about future performance. This lens is particularly relevant given the study’s focus on whether pre-admission metrics truly signal readiness for academic success in demanding science courses. Second, the study draws on Cummins’ (33) theory of Cognitive Academic Language Proficiency (CALP), which differentiates between basic interpersonal communication skills and the advanced linguistic competencies needed for academic tasks. From this perspective, English proficiency—especially in reading comprehension and discipline-specific vocabulary—is not just a support skill but a core predictor of success in science-heavy curricula like physics. Together, these frameworks underscore the importance of examining both cognitive test scores and language skills as integrated predictors of academic performance in health science education.

This study contributes to the literature by shifting focus from general GPA or long-term academic outcomes to a single, critical science course: introductory physics. As a cognitively demanding and language-dependent subject, physics offers a focused lens for examining how well current admission criteria predict academic readiness during the early stages of medical training. By doing so, it addresses a clear gap in both local and international research and offers practical implications for refining admission strategies in Saudi health science education.

## 2 Research questions

This study investigates the predictive validity of the General Aptitude Test (GAT), the Scholastic Achievement Admission Test (SAAT), and English language performance in relation to students’ performance in physics courses, which serve as a prerequisite for medical studies. The research questions guiding this study are as follows:

1. Individual Predictors of Physics Performance: Do GAT scores, SAAT scores, English course averages, reading proficiency test scores, and communication proficiency test scores individually predict students’ performance in physics? If so, which predictor demonstrates the strongest association with physics performance, and which is the weakest?
2. Combined Predictors of Physics Performance: When GAT scores, SAAT scores, English course averages, reading proficiency test scores, and communication proficiency test scores are combined in a multivariate regression analysis, what percentage of variance in physics performance can be explained by these predictors?

## 3 Research method

To provide context for this study, it is important to clarify the educational background of the participants, the focus on physics courses, the measurement of English language competence, and the analytical methods used.

### 3.1 Participants and context

This study employed a convenience sampling approach, utilizing academic data from 250 male freshmen enrolled in the preparatory year at a public health sciences university in Saudi Arabia. Participants were selected based on the following inclusion criteria: (1) completion of both semesters of the preparatory program, including English and science courses; (2) availability of complete records for GAT, SAAT, and English proficiency scores; and (3) completion of the final exam in the physics course. Students who withdrew, transferred, or had missing data were excluded from the analysis.

During the first semester, students undertook intensive English language instruction in grammar, reading, and communication. In the second semester, they progressed to foundational science subjects, including biology, chemistry, and physics. At the end of the academic year, students were ranked by cumulative GPA and competed for placement in selective health-related majors—such as medicine, dentistry, pharmacy, and applied medical sciences—each with its own GPA threshold. Instruction throughout the preparatory year was conducted in English.

### 3.2 Physics course overview

The introductory physics course served as a foundational requirement for all medical-track students. Its objective was to develop a comprehensive understanding of essential physics concepts relevant to the medical field. By the end of the course, students were expected to demonstrate competence in areas such as kinematics and dynamics, wave mechanics, properties of matter, electricity and magnetism, electromagnetism, optics, and selected topics in modern physics, including atomic and nuclear applications. The course was taught in English and delivered through 4 h of lecture per week in large classrooms. Student assessment consisted of two midterm exams and one cumulative final exam.

### 3.3 Measurement of English competence

English language competence was assessed using three indicators: (1) the average grade from students’ first-semester English courses, (2) a reading proficiency test, and (3) a communication proficiency test. The English course average reflected performance across integrated language instruction modules, while the two standardized tests provided focused assessments of academic reading and communication skills.

Both tests were designed by a panel of senior English instructors at the university and underwent a formal validation process. Content validity was established through expert review to ensure that the items aligned with course objectives and academic demands in science learning. Reliability was assessed using Cronbach’s alpha, yielding internal consistency values. The tests were piloted with 30 students from a previous cohort, and item analysis was conducted to assess difficulty and discrimination. Based on the results and instructor feedback, revisions were made to improve clarity and alignment with learning outcomes.

- Reading Proficiency Test: This test evaluated students’ ability to comprehend academic and general texts. It included tasks related to main idea identification, inferencing, referencing, interpretation of diagrams, distinguishing between fact and opinion, and analyzing cause-and-effect relationships. The test also measured academic vocabulary and critical reading strategies.
- Communication Proficiency Test: This test assessed students’ written communication abilities, including their use of rhetorical modes such as comparison, definition, and argumentation. It evaluated students’ skills in paraphrasing, summarizing, predicting outcomes, and organizing coherent paragraphs using topic sentences and supporting evidence.

### 3.4 Data analysis

Prior to analysis, the dataset was screened for missing values and data quality. Records with incomplete variables were excluded. Descriptive statistics were computed to examine variable distributions. Outlier analysis was conducted using standardized residuals and Cook’s distance. No cases exceeded the threshold for undue influence and were therefore retained in the dataset.

Statistical analyses were conducted using. Simple linear regression was used to examine the individual predictive strength of each independent variable—GAT scores, SAAT scores, English course averages, reading test scores, and communication test scores—on students’ final grades in the physics course. Multiple regression analysis was then performed to assess the combined predictive power of all variables. Statistical significance was determined at the *p* < 0.05 level.

## 4 Results

To address the first research question, which aimed to examine the individual predictive power of each variable, the study employed simple linear regression analysis. Table 1 presents the variance explained by each predictor (R^2^) and its statistical significance in relation to students’ physics course grades. Among the individual predictors, the reading proficiency test emerged as the strongest predictor, accounting for 19.6% of the variance in physics performance. The English course average was the second strongest predictor, explaining 14.7% of the variance. The General Aptitude Test (GAT) and the Scholastic Achievement Admission Test (SAAT) explained 9.4 and 7.9% of the variance, respectively. In contrast, the communication proficiency test was the weakest predictor, explaining only 7.2% of the variance in physics grades.

**Table 1:** Individual summary to predict physics grade.

Model	Variable	R	R square	Adjusted R square	Regression coefficient	Std. error	T	Coefficient *p*-value
1	GAT	0.306	0.094	0.090	0.051	0.010	5.100	0.000
2	SAAT	0.281	0.079	0.075	0.047	0.010	4.700	0.000
3	English_average	0.383	0.147	0.143	0.729	0.116	6.284	0.000
4	Reading_test	0.443	0.196	0.193	0.416	0.054	7.704	0.000
5	Communication_test	0.268	0.072	0.068	0.292	0.068	4.294	0.000

When the predictors were combined in a multivariate regression model, the explanatory power increased significantly. As shown in Table 2, the combined model explained 29.3% of the variance in physics course grades, demonstrating that the integration of multiple predictors provides a more robust prediction of academic performance than any single predictor alone.

**Table 2:** Combined model summary.

Model	Variable	R	R square	Adjusted R square	Std. error of the estimate
1	CT, GAT, SAAT, ENGL, RT	0.542	0.293	0.278	0.736

Predictors: (Constant). Communication test (CT), SAAT, GAT, English average (ENGL), Reading test (RT).

Further analysis of the regression coefficients, as presented in Table 3, revealed that the GAT and the reading proficiency test were the most statistically significant predictors, both with a *p*-value of 0.000. The English course average also showed statistical significance, with a p-value of less than 0.05. Based on the coefficients, the regression equation for predicting students’ performance in physics was derived as follows:


$$\begin{array}{l} \text{Physics Grade}=-2.902+0.034(\mathrm{GAT})+0.019(\text{SAAT})\\ +0.327(\text{English Average})+0.277(\text{Reading Test})\\ –0.031(\text{Communication Test}). \end{array}$$

**Table 3:** Coefficient of each predictor.

Model		Unstandardized coefficients		Standardized coefficients	*t*	Sig.	Coefficients^a^
		B	Std. error	Beta			
1	(Constant)	−2.902	1.056		−2.748	0.006	
	GAT	0.034	0.010	0.201	3.257	0.001	*p* < 0.0005
	SAAT	0.019	0.010	0.115	1.853	0.065	
	English_average	0.327	0.128	0.172	2.562	0.011	*p* < 0.05
	Reading_test	0.277	0.074	0.296	3.755	0.000	*p* < 0.0005
	Communication_test	−0.031	0.081	−0.028	−0.379	0.705	

a Dependent variable: physics grade.

This equation indicates that higher scores on the GAT, SAAT, English course average, and reading proficiency test are associated with better performance in physics, while the communication proficiency test had a negligible negative effect on the prediction. These findings highlight the relative importance of cognitive abilities (as measured by the GAT), subject-specific knowledge (as measured by the SAAT), and language proficiency (particularly reading skills) in predicting academic success in physics.

## 5 Discussion

The selection of students for medical and health science programs is a complex process that requires careful consideration of both cognitive and non-cognitive factors. This study examined the predictive validity of the General Aptitude Test (GAT), the Scholastic Achievement Admission Test (SAAT), and English language competence in relation to students’ performance in physics. The findings revealed that while the GAT and SAAT demonstrated moderate predictive power, explaining 9.4 and 7.9% of the variance in physics grades, respectively, the reading proficiency test emerged as the strongest individual predictor, accounting for 19.6% of the variance. These results highlight the importance of cognitive abilities and language skills in academic success but also underscore the limitations of traditional admission criteria in fully capturing the range of competencies required for success in university-level science courses.

### 5.1 Predictive value of standardized admission tests

The predictive value of standardized admission tests like the GAT and SAAT remains a topic of debate in educational institutions worldwide. While some studies have reported strong correlations between these tests and academic performance, others have found inconsistent or weak associations (11, 25). For example, a study found a strong correlation between academic performance and scores on the SAAT, GAT, and high school final grades, with the SAAT showing the strongest predictive power (8). However, this study was limited by its small sample size (*n* = 91). In contrast, a larger study involving 737 students found that the SAAT was a positive predictor of cumulative GPA (cGPA) during the pre-clinical years but not during the clinical years, while the GAT showed no significant predictive value (11). These inconsistencies suggest that the predictive power of standardized tests may vary depending on the stage of education and the specific demands of the program.

In the context of this study, the GAT and SAAT demonstrated moderate predictive power, reflecting their role in assessing general cognitive abilities and subject-specific knowledge, respectively. The GAT, which measures skills such as critical thinking and problem-solving, was a significant predictor of physics performance, likely because these skills are essential for understanding and applying complex physics concepts. Similarly, the SAAT, which assesses knowledge in areas such as physics, chemistry, and mathematics, also showed predictive value, underscoring the importance of foundational scientific knowledge for success in physics courses. However, the relatively low percentage of variance explained by these tests suggests that other factors, such as language proficiency, motivation, and non-cognitive skills, may also play a critical role in academic performance.

### 5.2 The role of language proficiency

The inclusion of English language proficiency measures in this study provided valuable insights into the factors influencing physics performance. The reading proficiency test emerged as the strongest individual predictor, explaining 19.6% of the variance in physics grades. This finding aligns with the broader literature emphasizing the importance of language skills in academic settings, particularly in programs where instruction is delivered in English (26–30, 34). The ability to comprehend and analyze complex texts is essential for understanding physics concepts, which are often presented in dense and technical language. In contrast, the communication proficiency test, which assessed verbal communication skills, had a weaker predictive value, explaining only 7.2% of the variance. This may reflect the fact that physics courses typically emphasize quantitative reasoning and problem-solving over verbal communication, although the latter remains important for other aspects of medical education, such as patient interaction and teamwork.

### 5.3 Misalignment between high school and university education

The relatively weak association between standardized admission tests and academic performance in this study may also be attributed to the significant differences between high school and university teaching methods and assessment strategies. In high school, the emphasis is often on rote memorization and the recall of factual knowledge, which aligns with the design of many standardized tests. However, university programs, particularly in fields like physics and health sciences, prioritize higher-order cognitive skills such as critical thinking, problem-solving, and the application of knowledge to practical and clinical scenarios (26, 27). These competencies are not typically assessed by traditional eligibility tests like the GAT and SAAT, which may explain their limited predictive power in this study. For example, while the GAT measures general cognitive abilities such as logical reasoning and problem-solving, it does not fully capture the analytical and synthesis skills required to excel in physics, where students must apply theoretical concepts to solve complex problems. Similarly, the SAAT, which focuses on subject-specific knowledge, may not adequately assess the ability to integrate and apply this knowledge in new contexts, a skill that is critical for success in university-level science courses.

### 5.4 The importance of non-academic competencies

In addition to cognitive and language skills, non-academic competencies such as leadership, communication, teamwork, and community engagement are increasingly recognized as essential for success in medical and health science programs (19). These competencies are typically developed during university education rather than in high school and are not adequately assessed by traditional admission tests. In the context of this study, the inclusion of English language proficiency measures provided some insight into the role of these skills in academic performance. However, the relatively low predictive power of the communication proficiency test suggests that while language proficiency is important, the specific skills assessed by this test may be less relevant to performance in physics, which emphasizes quantitative reasoning and problem-solving over verbal communication.

### 5.5 Implications for admission processes

The findings of this study have important implications for the design of admission processes in medical and health science programs. While the GAT, SAAT, and reading proficiency test were significant predictors of physics performance, the relatively low variance explained by the combined model (29.3%) suggests that additional factors—such as non-cognitive skills, motivation, and study habits—should also be considered in the admission process. This is particularly relevant in the context of competency-based education, which emphasizes the development of both cognitive and non-cognitive skills, such as clinical reasoning, ethical decision-making, and interpersonal communication (11, 21). Future research could explore the role of these factors in academic success, as well as the potential benefits of incorporating non-cognitive assessments like the multiple mini-interview (MMI) into the admission process. By adopting a more holistic approach to student selection, medical and health science programs can better identify candidates who are not only academically prepared but also possess the personal and professional qualities needed for success in healthcare professions.

## 6 Limitations of the study

While this study provides valuable insights into the predictive validity of standardized admission tests (GAT, SAAT, and English competence measures) on students’ performance in an introductory physics course, several limitations must be acknowledged. First, the sample size of 250 students, although sufficient for initial analysis, may not fully represent the diversity of academic abilities, socioeconomic backgrounds, and educational preparation across Saudi Arabia. This limited sample restricts the generalizability of the findings to the broader population of Saudi college students.

Second, the study focused exclusively on performance in a single introductory physics course as the outcome variable. Physics, as a subject, may require specific cognitive skills (e.g., mathematical reasoning and problem-solving) that differ from other disciplines, potentially limiting the applicability of these findings to other academic fields. The low explained variance (29.3%) in the multivariate regression model suggests that additional unexamined factors—such as students’ prior physics exposure, motivation, study habits, or instructor effectiveness—may play significant roles in academic performance but were not accounted for in this analysis.

Third, the reliance on GAT, SAAT, and English proficiency measures as predictors assumes these tools comprehensively capture the skills necessary for success in higher education. However, these standardized tests may not fully assess critical non-cognitive factors (e.g., resilience, time management, or collaborative skills) or contextual variables (e.g., high school quality or access to preparatory resources), which could influence physics performance. Additionally, the English competence measures, while significant, may reflect proficiency in a second language rather than mastery of physics-specific terminology or conceptual understanding, introducing potential measurement bias.

Finally, the study’s design did not account for longitudinal effects. Analyzing performance in a single course at one point in time overlooks how students’ abilities might evolve over their academic careers or how admission criteria predict long-term outcomes, such as degree completion or overall GPA. These limitations collectively suggest that while GAT, SAAT, and English proficiency are statistically significant predictors, their practical utility in explaining physics performance remains constrained, underscoring the need for a more comprehensive national study.

## 7 Conclusion

This study set out to evaluate the extent to which standardized admission tests—namely the General Aptitude Test (GAT), the Scholastic Achievement Admission Test (SAAT), and English language proficiency measures—can predict students’ performance in an introductory physics course, a critical foundation for medical and health science education. The results offer a nuanced perspective: while GAT, SAAT, and reading proficiency individually hold moderate predictive value (explaining 9.4, 7.9, and 19.6% of the variance in physics grades, respectively), their combined explanatory power remains limited, accounting for only 29.3% of the variance. These findings illuminate both the strengths and shortcomings of current admission criteria in Saudi Arabia, prompting a deeper reflection on how well these tools align with the demands of university-level science education and the broader goals of medical training.

The moderate predictive power of GAT and SAAT underscores their relevance in assessing foundational cognitive abilities and subject-specific knowledge. The GAT’s focus on critical thinking and problem-solving aligns with the intellectual demands of physics, where students must grapple with abstract concepts and apply logical reasoning to solve problems. Likewise, the SAAT’s emphasis on scientific knowledge in areas like physics and mathematics provides a baseline for academic readiness. However, the modest variance explained by these tests suggests they capture only a fraction of what contributes to success in a university setting. This partial picture may stem from their design, which mirrors high school learning environments that prioritize rote memorization and factual recall over the analytical and integrative skills emphasized in higher education. Physics, as a discipline, requires not just knowledge but the ability to synthesize and apply it—a competency that standardized tests like GAT and SAAT may not fully measure. This misalignment between high school preparation and university expectations highlights a structural challenge in the transition to higher education, one that admission processes must address to better prepare students for academic rigor.

The standout role of reading proficiency, explaining nearly 20% of the variance, adds another layer of insight. In an academic context where English serves as the medium of instruction, the ability to comprehend complex, technical texts is indispensable for mastering physics concepts. This finding resonates with prior research underscoring language proficiency as a cornerstone of academic success in science programs delivered in a second language (26, 27). Yet, the weaker predictive value of communication proficiency (7.2% variance) suggests that not all language skills are equally relevant to physics performance. While reading enables students to decode and analyze course material, verbal communication may play a lesser role in a subject centered on quantitative reasoning. This distinction invites a broader question: how should admission criteria weigh different facets of language competence, especially in programs like medicine, where communication becomes vital in clinical settings? The prominence of reading proficiency in this study signals a need to prioritize language skills in student selection, but it also reveals the limitations of relying solely on cognitive and linguistic measures without considering their context-specific relevance.

Beyond these measurable factors lies a critical gap: the 70.7% of variance in physics grades left unexplained by the combined model. This substantial shortfall points to the influence of unexamined variables—motivation, study habits, prior exposure to physics, and non-cognitive skills like resilience and teamwork—that likely shape academic outcomes. In medical and health science education, where success hinges on more than intellectual ability, these non-academic competencies are particularly significant. Leadership, ethical reasoning, and interpersonal skills, which standardized tests do not assess, are essential for future healthcare professionals (19). The low predictive power of the current model thus reflects not a failure of GAT, SAAT, or English proficiency as tools, but rather their inability to capture the full spectrum of attributes required for success in physics and beyond. This limitation aligns with global debates about the adequacy of standardized testing in predicting university performance, where findings range from strong correlations to weak associations depending on context and program demands (11, 25).

For Saudi policymakers and educators, these results carry practical implications. The modest explanatory power of existing admission criteria suggests that while they serve a purpose, they fall short of providing a comprehensive basis for selecting students likely to thrive in medical and health science programs. A more holistic approach—integrating cognitive assessments with measures of non-cognitive skills, such as through tools like the multiple mini-interview (MMI)—could bridge this gap. Such a shift would align with competency-based education models that value clinical reasoning, ethical decision-making, and collaboration alongside academic prowess (21). Moreover, the reliance on English proficiency as a key predictor underscores the need to strengthen language preparation in high school curricula, ensuring students enter university equipped to engage with technical material. However, implementing these changes requires more than localized adjustments; it demands a national study with a larger, more diverse sample to validate these findings and explore additional predictors. Only through such evidence-based research can Saudi Arabia refine its admission system to better serve its students and healthcare sector.

In conclusion, this study reveals that while GAT, SAAT, and reading proficiency offer valuable insights into students’ potential in physics, they are but pieces of a larger puzzle. Their limited combined predictive power reflects the complexity of academic success, which transcends test scores and language skills to encompass a broader array of personal and contextual factors. By highlighting these gaps, the research not only contributes to the ongoing discourse on admission criteria but also calls for a reevaluation of how Saudi Arabia prepares and selects its future medical professionals. Moving forward, a balanced, evidence-driven approach—one that bridges high school and university expectations while embracing both cognitive and non-cognitive dimensions—holds the promise of fostering a more capable and well-rounded student body, ready to meet the challenges of science education and healthcare practice.

## Data Availability

The raw data supporting the conclusions of this article will be made available by the authors, without undue reservation.
